# Pancreatic Adenocarcinoma Up-Regulated Factor Promotes Epithelial–Mesenchymal Transition and Lung Metastasis in Hepatocellular Carcinoma

**DOI:** 10.3390/ijms27146213

**Published:** 2026-07-12

**Authors:** Jeong-Ran Park, Hyejun Ham, Miso Lee, Jae Ho Seo, Dong-Keon Lee

**Affiliations:** 1 Division of Research Program, Scripps Korea Antibody Institute, Chuncheon-Si 24341, Gangwon-Do, Republic of Korea; pjr@skai.or.kr (J.-R.P.);; 2 Department of Biochemistry, School of Medicine, Wonkwang University, Iksan-Daero 460, Iksan-Si 54538, Jeonbuk-Do, Republic of Korea; bionian9@wku.ac.kr; 3 Sarcopenia Total Solution Center, School of Medicine, Wonkwang University, Iksan-Daero 460, Iksan-Si 54538, Jeonbuk-Do, Republic of Korea; 4 Institute of Wonkwang Medical Science, School of Medicine, Wonkwang University, Iksan-Daero 460, Iksan-Si 54538, Jeonbuk-Do, Republic of Korea

**Keywords:** PAUF, hepatocellular carcinoma, HepG2, Huh-7, TGF-β, TGF-βRI/II, Smads, MEK-ERK signaling pathway, epithelial–mesenchymal transition, lung metastasis

## Abstract

Pancreatic adenocarcinoma upregulated factor (PAUF), a novel secreted protein highly expressed in pancreatic ductal adenocarcinoma, also influences cell invasiveness, motility, and proliferation in several cancer types. Transforming growth factor-beta (TGF-β)-induced PAUF expression enhances cancer cell migration and invasion in pancreatic ductal adenocarcinoma through mitogen-activated protein kinase (MEK)–extracellular signal-regulated kinase (ERK) activation; however, the roles of PAUF in regulating epithelial–mesenchymal transition (EMT) and promoting lung metastasis in hepatocellular carcinoma (HCC) remain unclear. Thus, we investigated the regulatory mechanisms and functional roles of TGF-β-induced PAUF expression in the HCC cell lines HepG2 and Huh-7, which showed high and low expression of intact TGF-β type I and II receptors, respectively. We found that TGF-β-induced PAUF expression is mediated through the activation of the TGF-β type I/II receptor–Smads signaling pathway and that PAUF promotes EMT-associated migration and invasion by stimulating the MEK–ERK signaling cascade. In vivo studies further demonstrated that PAUF plays a critical role in lung metastatic potential, as PAUF knockdown HepG2 cells exhibited markedly reduced pulmonary metastasis, whereas PAUF-overexpressing Huh-7 cells showed substantially enhanced lung metastasis. This study identifies PAUF as a critical promoter of lung metastatic potential in HCC cells and a potential therapeutic target for HCC.

## 1. Introduction

Hepatocellular carcinoma (HCC) is the fifth most common malignancy and third leading cause of cancer-related mortality worldwide [1]. Despite advances in diagnostic technologies and therapeutic strategies, rates of early diagnosis and long-term survival remain poor for patients with HCC [2]. Clinically, HCC is intrinsically metastatic, exhibiting pronounced aggressiveness, strong intrahepatic invasiveness, and high rates of postoperative recurrence; this fundamental biological hallmark is the main cause of therapeutic failure and cancer-related mortality [3,4]. Epithelial–mesenchymal transition (EMT) is a reversible biological process through which epithelial cells acquire mesenchymal phenotypes under specific conditions [5]. This transition is accompanied by the loss of epithelial polarity and cell–cell adhesion, cytoskeletal reorganization, and the acquisition of enhanced migratory and invasive properties [5,6,7]. Accordingly, EMT is recognized as a key event in the metastatic progression of various cancers, including HCC [8,9].

This phenotypic conversion is driven by EMT-inducing transcription factors, including SNAI1, SLUG, and ZEB family proteins, which downregulate epithelial markers (E-cadherin, ZO-1, claudin, and cytokeratin) and upregulate mesenchymal markers (N-cadherin, fibronectin, and vimentin). These processes increase the migratory and invasive capacities of cancer cells [10].

Transforming growth factor-beta (TGF-β) is a potent inducer of EMT during cancer progression [11]. TGF-β is a pleiotropic cytokine that regulates diverse cellular processes, including proliferation, differentiation, wound healing, immune modulation, and cancer progression [12,13]. Canonical TGF-β signaling is primarily mediated through Smads transcription factors. Following ligand binding, TGF-β engages TGF-βRII, which recruits TGF-βRI to form an active receptor complex. TGF-βRII then transphosphorylates TGF-βRI at serine and threonine residues within the juxtamembrane glycine-serine-rich (GS) domain, thereby activating TGF-βRI kinase activity. Activated TGF-βRI subsequently phosphorylates Smad2/3, initiating downstream canonical TGF-β signaling [14,15]. Phosphorylated Smad2/3 then interacts with cytoplasmic Smad4 to form a heteromeric Smads complex, which subsequently translocates into the nucleus to regulate the transcription of TGF-β-responsive target genes [16,17]. This canonical TGF-β pathway exerts tumor-suppressive effects in cancer cells by transcriptionally upregulating genes associated with cell cycle arrest (such as p21) and apoptosis (such as *Bim*) [18]. In addition to canonical Smad-dependent signaling, TGF-β can activate several non-canonical signaling pathways, including mitogen-activated protein kinase pathways, such as c-Jun N-terminal kinase, p38, MEK, and extracellular signal-regulated kinases 1/2, as well as the phosphatidylinositol 3-kinase/Akt pathway [19,20]. These Smad-independent signaling cascades are closely associated with cancer progression and aggressive tumor phenotypes. Among them, the MEK–ERK pathway has been implicated as a key mediator of TGF-β-driven EMT during cancer progression [21] and is also involved in liver cancer progression and metastasis [22,23]. Collectively, TGF-β-mediated Smads and non-Smads signaling pathways exert dual and often contradictory effects on tumor growth and metastasis.

Pancreatic adenocarcinoma upregulated factor (PAUF), also known as ZG16B, is a tumor-associated secretory protein that contributes to cancer progression. Initially identified as a gene highly upregulated in pancreatic ductal adenocarcinoma (PDAC), PAUF has also been associated with the malignant progression of other solid tumors, including ovarian cancer, colorectal cancer, and cervical cancer [24,25,26]. In pancreatic cancer, PAUF promotes tumorigenesis by regulating the tumor microenvironment through autocrine and paracrine mechanisms [27]. PAUF has been shown to bind toll-like receptor 4 (TLR4), leading to TLR4-mediated PDAC cell migration and invasion, suggesting that PAUF may facilitate carcinogenesis and metastasis through activation of TLR4-dependent signaling [28]. Recently, we showed that TGF-β-induced PAUF expression enhances cell migration and invasion through MEK–ERK signaling pathway activation in the PDAC cell line Panc-1, suggesting that PAUF may promote cancer metastasis by activating the TGF-β signaling pathway [29]. However, the biological functions of PAUF in regulating EMT processes and promoting lung metastasis in HCC have not been studied either in vitro or in vivo.

In this study, we investigated the regulatory effects and functional roles of TGF-β-induced PAUF expression in HepG2 cells (high expression of intact TGF-βRI/II) and Huh-7 cells (low expression of intact TGF-βRI/II) [30]. We found that TGF-β-induced PAUF expression was regulated through activation of the TGF-βRI/II–Smads signaling pathway, and promoted EMT-related cell migration and invasion via activation of the MEK–ERK signaling cascade. We also assessed the PAUF-mediated lung metastatic potential of HCC cells in vivo. PAUF-knockdown HepG2 cells exhibited markedly lower lung metastatic potential than control HepG2 cells, whereas PAUF-overexpressing Huh-7 cells showed markedly higher lung metastatic potential than control Huh-7 cells.

Overall, our findings suggest that PAUF plays a crucial role in promoting the lung metastatic potential of HCC cells, and may represent a novel therapeutic target for HCC.

## 2. Results

### 2.1. Evaluation of TGF-β-Induced PAUF Expression and Cell Motility in the Human Hepatocellular Carcinoma (HCC) Cells

**Figure 1:**
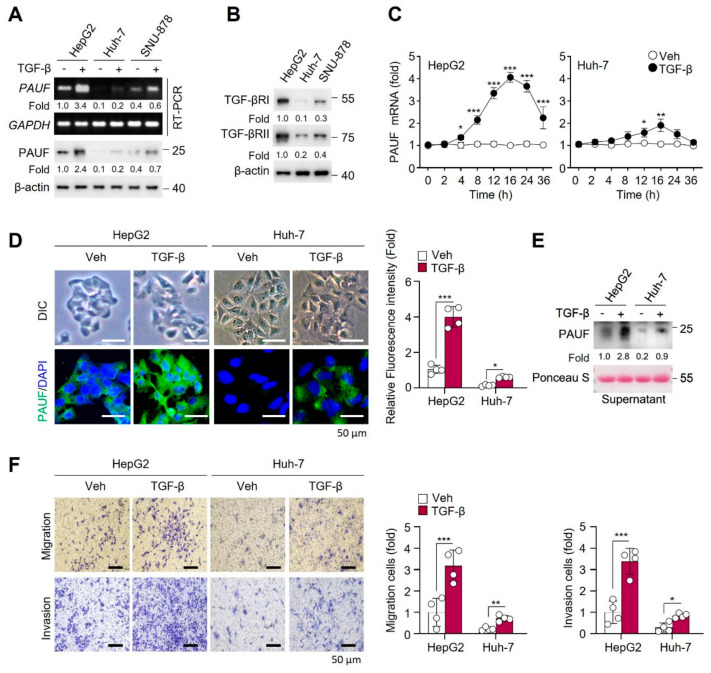
TGF-β-induced pancreatic adenocarcinoma upregulated factor (PAUF) expression promotes the metastatic potential of human hepatocellular carcinoma (HCC) cells. (**A**) HCC cell lines (HepG2, Huh-7, SNU-878) treated with vehicle or TGF-β (10 ng/mL) for 12 h. Levels of PAUF expression was determined by RT-PCR and immunoblotting. (**B**) Basal expression levels of TGF-βRI and TGF-βRII were analyzed by immunoblotting. (**C**) PAUF expression in HepG2 and Huh-7 cells stimulated with vehicle or TGF-β for the indicated time periods, analyzed by qRT-PCR (*n* = 3). (**D**) Changes in cell morphology and intracellular PAUF expression levels in HepG2 and Huh-7 cells treated with vehicle or TGF-β for 24 h, observed using an optical microscope (magnification, 100×) and immunofluorescence staining (magnification, 400×) with Alexa 488-labeled anti-PAUF antibody, respectively; nuclei were stained with DAPI. Scale bar, 50 μm. PAUF-expressing cells were quantified in four fields per sample using ImageJ software (*n* = 4). (**E**) Levels of secreted PAUF in culture supernatants, measured using immunoblotting and ponceau-S staining. (**F**) Cell migration and invasion examined by transwell migration and Matrigel invasion assays, quantified in four fields per sample (*n* = 4). Scale bar, 50 μm; magnification, 40×. PAUF Statistical significance in (**C**,**D**,**F**) was calculated using two-way ANOVA followed by *post hoc* multiple comparisons test with Bonferroni correction. Data are presented as the mean ± standard deviation (SD). * *p* < 0.05, ** *p* < 0.01, *** *p* < 0.001.

First, we examined whether TGF-β regulates PAUF expression in three different HCC cell lines: HepG2, Huh-7, and SNU-878. HepG2 cells exhibited the highest levels of PAUF mRNA and protein expression, whereas Huh-7 cells showed the lowest levels under both basal and TGF-β-treated conditions (Figure 1A). To determine whether TGF-β-induced differential PAUF expression is associated with the basal expression levels of TGF-βRI and -βRII (TGF-βRI/II) in HCC cell lines, we confirmed the basal expression levels of TGF-βRI/II in HepG2, Huh-7, and SNU-878 cells. As expected, the expression levels of intact TGF-βRI/II were highest in HepG2 cells and lowest in Huh-7 cells (Figure 1B). Next, we evaluated the mRNA expression levels of PAUF in TGF-β-stimulated HepG2 and Huh-7 cells at different time points. TGF-β treatment significantly increased PAUF mRNA levels in HepG2 cells after 8 h compared with vehicle treatment; this increase was further enhanced at 16 h, whereas Huh-7 cells showed lower levels of PAUF mRNA expression than HepG2 cells (Figure 1C). HepG2 cells exhibited higher expression levels of PAUF intra- and extracellular proteins than Huh-7 cells under both basal and TGF-β-treated conditions (Figure 1D,E). Moreover, HepG2 cells exhibited more substantial EMT-like morphological changes and increased cell motility compared to Huh-7 cells under both TGF-β-treated and TGF-β-untreated conditions (Figure 1D,F). These findings suggest that PAUF promotes the metastatic potential of HCC cells.

### 2.2. TGF-β Induces PAUF Expression via Activation of the TGF-βRI/II-Smad2/3 Pathway in HepG2 and Huh-7 Cells

**Figure 2:**
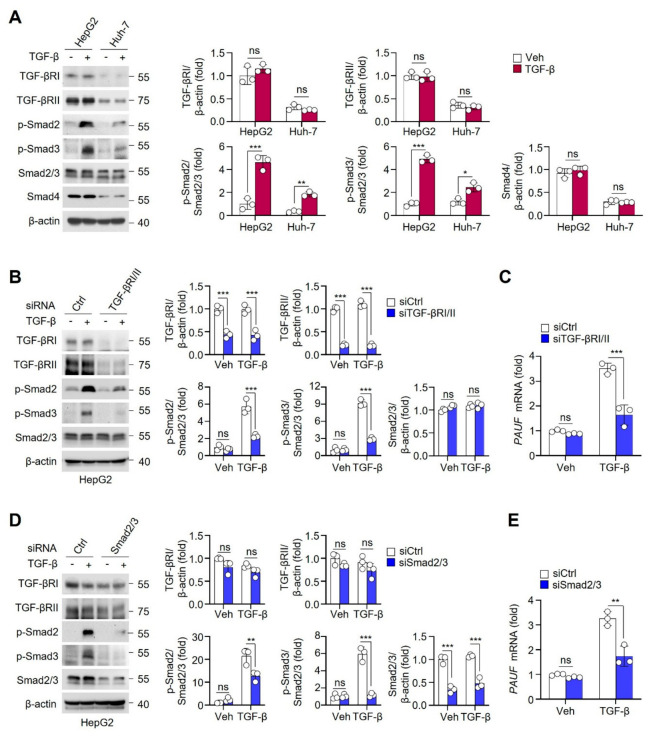

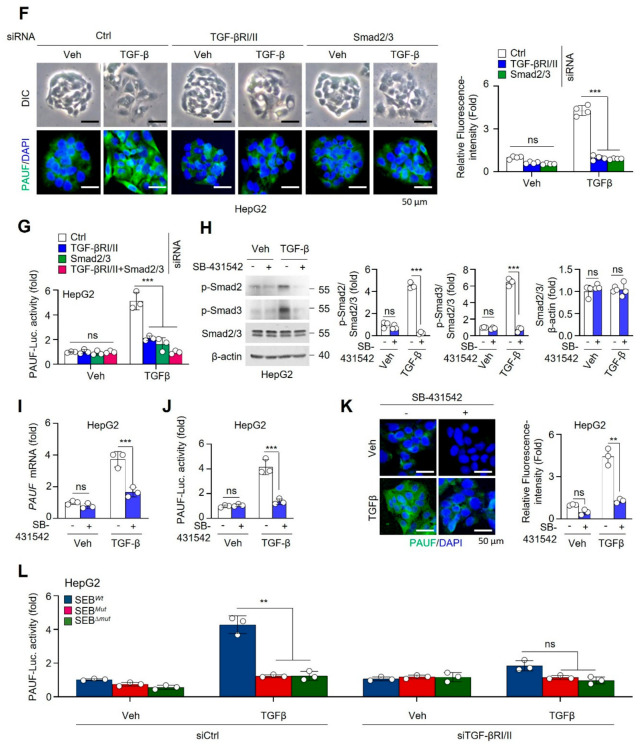
Inhibition of TGF-βR–Smad signaling suppresses TGF-β-induced PAUF expression in HepG2 cells. (**A**) Comparison of protein and phosphorylation levels of TGF-β–mediated TGF-βRI/II–Smads signaling molecules in the human hepatocellular carcinoma (HCC) cell lines HepG2 and Huh-7. HepG2 and Huh-7 cells were stimulated with vehicle or TGF-β (10 ng/mL) for 1 h. Protein and phosphorylation levels of indicated signaling molecules analyzed by immunoblotting. HepG2 cells were transfected with 100 nM scrambled (Ctrl), (**B**,**C**) TGF-βRI/II or (**D**,**E**) Smad2/3 siRNAs, then treated with vehicle or TGF-β (10 ng/mL) for (**B**,**D**) 1 h or (**C**,**E**,**F**) 24 h. (**B**,**D**) TGF-βRI/II expression and Smad2/3 phosphorylation determined by immunoblotting. (**C**,**E**) Relative mRNA levels of PAUF analyzed by qRT-PCR (*n* = 3). (**F**) Cell morphological changes and intracellular PAUF expression visualized by an optical microscope (magnification, 100×) and immunofluorescence staining (magnification, 400×) using an Alexa 488-labeled anti-PAUF antibody, respectively; nuclei were counterstained with DAPI. Scale bar, 50 μm. Relative number of PAUF-expressing cells was quantified in four randomly selected fields per sample using ImageJ software (*n* = 4). (**G**) Promoter activity in HepG2 cells transfected with the indicated siRNA alone or in combination with the PAUF promoter–Luc vector (pLuc–PAUF), then treated with vehicle or TGF-β for 24 h, measured using a luminometer in cell lysates (*n* = 3). HepG2 cells were pretreated with SB-431542 (10 μM) for 1 and stimulated with vehicle or TGF-β for (**H**) 1 or (**I**) 24 h. (**H**) Smad2/3 phosphorylation was assessed by immunoblotting; (**I**) PAUF mRNA expression was analyzed by qRT-PCR (*n* = 3). (**A**,**B**,**D**,**H**) Densitometry was performed using Image J software (*n* = 3). (**J**) Promoter activity in HepG2 cells transfected with pLuc–PAUF and treated with vehicle or TGF-β 24 h after pretreatment with SB-431542 (10 μM) for 1 h, measured using a luminometer in cell lysates (*n* = 3). (**K**) HepG2 cells were pretreated with SB-431542 (10 μM) for 1 h, followed by stimulation with vehicle or TGF-β (10 ng/mL) for 24 h. Immunofluorescence staining was performed as described in upper panel, F. Scale bar, 50 μm; magnification, 400×. (**L**) Promoter activity in HepG2 cells transfected with scramble (Ctrl) or TGF-βRI/II siRNAs together with PAUF promoter–Luc vectors (SBE*^WT^*) or PAUF mutant promoter–Luc vectors (SBE*^Mut^*, SBE*^∆mut^*), then stimulated with vehicle or TGF-β for 24 h, measured using a luminometer in cell lysates (*n* = 3). Statistical significance was determined using two-way ANOVA followed by *post hoc* multiple comparisons test with Bonferroni correction. Data are presented as the mean ± SD. ns, not statistically significant, * *p* < 0.05, ** *p* < 0.01, *** *p* < 0.001.

**Figure 3:**
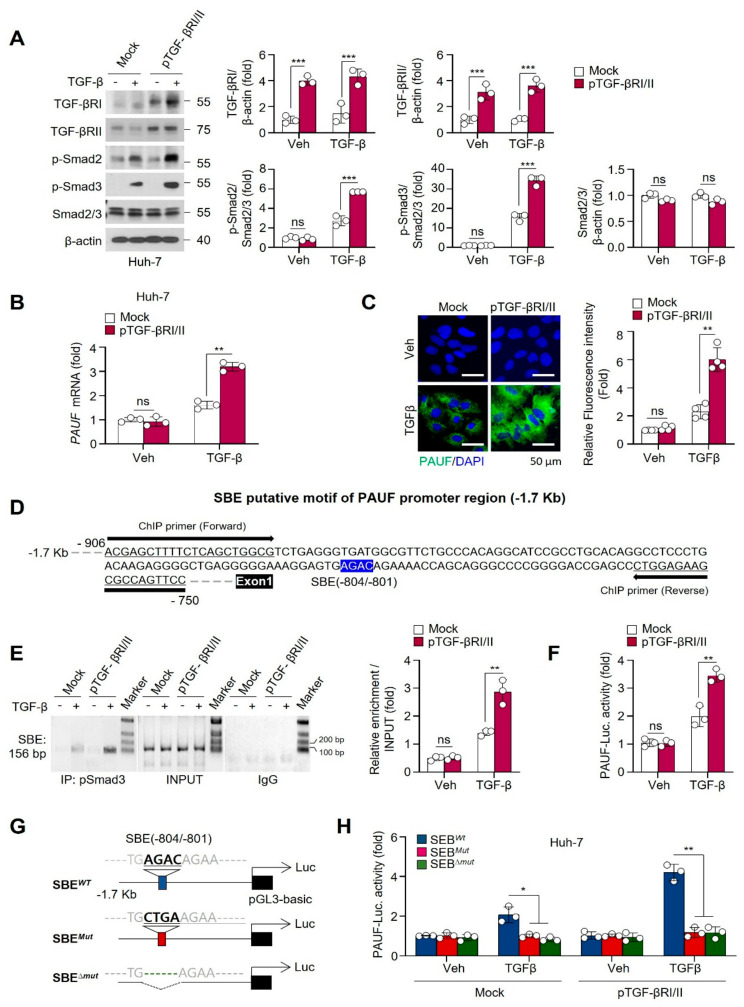
Ectopic expression of TGF-β receptor I/II (TGF-βRI/II) augments the TGF-β-induced increase in PAUF expression in Huh-7 cells. Huh-7 cells were transfected with control vector pCMV6 (Mock) or TGF-βRI/II expression vector (pTGF-βRI/II), then stimulated with vehicle or TGF-β (10 ng/mL) for (**A**) 1 or (**B**,**C**) 24 h. (**A**) Phosphorylation and protein levels of TGF-β-mediated TGF-βR/Smads signaling molecules determined by immunoblotting. Densitometry was performed using Image J software (*n* = 3). (**B**) PAUF mRNA expression analyzed by qPCR (*n* = 3). (**C**) Intracellular PAUF levels determined by immunofluorescence staining with an Alexa 488-labeled anti-PAUF antibody; nuclei were stained with DAPI. Scale bar, 50 µm; magnification, 400×. PAUF. Relative fluorescence intensities measured in four randomly selected fields per sample using ImageJ software (*n* = 4). (**D**) Schematic representation of the PAUF promoter (−1.7 kb), indicating the putative Smad-binding element (SBE; −804/−801) used for chromatin immunoprecipitation (ChIP) analysis and assessment of PAUF promoter activity. (**E**) Binding activity of pSmad3 to SBE within the PAUF promoter of Huh-7 cells transfected with Mock or pTGF-βRI/II vector and treated with TGF-β for 1 h, measured using the ChIP assay. ChIP enrichment at the SBE motif (−804/−801) was quantified as fold enrichment analysis and normalized to the input control (*n* = 3). (**F**) PAUF promoter activity in Huh-7 cells transfected with Mock or pTGF-βRI/II vector with the PAUF promoter–Luc vector, then stimulated with vehicle or TGF-β for 24 h, measured using a luminometer in cell lysates (*n* = 3). (**G**) Schematic diagram of the putative SBE motif within PAUF promoter (−1.7 Kb) with SBE mutants. (**H**) Promoter activity in Huh-7 cells transfected with Mock or pTGF-βRI/II together with PAUF promoter–Luc vectors (SBE*^WT^*, SBE*^Mut^* or SBE*^∆mut^*), then treated with vehicle or TGF-β for 24 h, measured using a luminometer in cell lysates (*n* = 3). Statistical significance was assessed using two-way ANOVA, followed by *post hoc* multiple comparisons test with Bonferroni correction. All data are presented as the mean ± SD. ns, not statistically significant, * *p* < 0.05, ** *p* < 0.01, *** *p* < 0.001.

Our recent study demonstrated that TGF-β induces PAUF expression in the PDAC cell line Panc-1 via activation of Smad signaling [29]. To investigate TGF-β-induced differential PAUF expression in the HCC cell lines HepG2 and Huh-7 (Figure 1A–D), we first confirmed the expression or phosphorylation levels of TGF-β-mediated TGF-βRI/II–Smads signaling molecules. The basal expression levels of TGF-βRI/II and Smad4 were higher in HepG2 cells than in Huh-7 cells under both TGF-β-treated and untreated conditions. As expected, TGF-β treatment induced a significant increase in Smad2/3 phosphorylation in HepG2 cells compared with Huh-7 cells (Figure 2A). Consistent with our results, those of a previous study demonstrated that basal expression of TGF-βRI/II and Smad4 proteins was high in HepG2 cells. In contrast, Huh-7 and Hep3B cells expressed very low levels of TGF-βRI/II and Smad4 proteins [30]. To assess whether TGF-β signaling mediators are involved in PAUF expression, we next examined the effects of TGF-βRI/II or Smad2/3 knockdown on PAUF expression in TGF-β-stimulated HepG2 cells. TGF-βRI/II or Smad2/3 knockdown HepG2 cells were successfully generated, as confirmed by immunoblotting (Figure 2B,D). Knockdown of TGF-βRI/II or Smad2/3 markedly reduced Smad2/3 phosphorylation, mRNA and intracellular protein levels of PAUF, and EMT-associated morphological transitions compared with siRNA control (Ctrl)-transfected HepG2 cells treated with TGF-β (Figure 2B–F). Additionally, to investigate whether knockdown of TGF-βRI/II or Smad2/3 regulates TGF-β-induced increases in PAUF transcriptional activity, HepG2 cells were transiently transfected with the indicated siRNA alone or in combination with the PAUF promoter-Luc vector and then stimulated with vehicle or TGF-β. TGF-β-induced PAUF promoter activity was effectively inhibited by single knockdown of TGF-βRI/II or Smad2/3, as well as by double knockdown of TGF-βRI/II and Smad2/3 (Figure 2G). As expected, knockdown of TGF-βRI/II or Smad2/3 alone, as well as the combined knockdown of TGF-βRI/II and Smad2/3, abolished the TGF-β-induced increase in pSmad3 binding to the Smad-binding element (SBE; −804/−801) within the −1.7 kb PAUF promoter (Figure 3D and Figure S1A). We next assessed the inhibitory effects of the TGF-βRI kinase inhibitor SB-431532 on TGF-β-induced PAUF expression through the activation of TGF-β/Smad signaling in HepG2 cells. TGF-β treatment significantly increased Smad2/3 phosphorylation, which was effectively inhibited by SB-431532 (Figure 2H). Furthermore, the inhibitor abolished TGF-β-induced increases in PAUF mRNA expression, PAUF promoter activity, pSmad3 binding enrichment at the SBE motif, and intracellular PAUF protein levels (Figure 2I–K and Figure S1B). To further assess whether TGF-β-activated Smad proteins directly regulate PAUF transcription in HepG2 and Huh-7 cells, we utilized PAUF promoter–Luc vectors (SBE*^WT^*, SBE*^Mut^*, SBE*^∆mut^*) constructed in our previous study (Figure 3G) [29]. TGF-β markedly increased the transcriptional activity of SBE within the PAUF promoter (SBE*^WT^*), but not that of SBE mutants (SBE*^Mut^*, SBE*^∆mut^*) in high-intact TGF-βRI/II-expressing HepG2 cells. Moreover, this increase was not observed in TGF-βRI/II-knockdown HepG2 cells treated with TGF-β (Figure 2L). Next, we performed gain-of-function experiments to restore the expression of TGF-βRI/II in Huh-7 cells using a TGF-βRI/II expression vector (pTGF-βRI/II). Ectopic expression of TGF-βRI/II was confirmed by immunoblotting (Figure 3A). TGF-βRI/II overexpression markedly increased Smad2/3 phosphorylation, PAUF mRNA and intracellular protein levels, pSmad3 binding enrichment at the SBE motif, and PAUF promoter activity compared with control vector (Mock)-transfected Huh-7 cells stimulated with TGF-β (Figure 3A–C,E,F). Moreover, TGF-β slightly increased the transcriptional activity of REDD1 promoter SBE*^WT^*, but not that of SBE mutants (SBE*^Mut^*, SBE*^∆mut^*) in low-intact TGF-βRI/II-expressing Huh-7 cells; this slight increase significantly recovered the transcriptional activity of the PAUF promoter (SBE*^WT^*), but not that of SBE mutants in pTGF-βRI/II-overexpressing Huh-7 cells stimulated with TGF-β (Figure 3H). Overall, these findings provide strong evidence that TGF-β upregulates PAUF through activation of the TGF-βRI/II–Smads signaling pathway in HCC cells.

### 2.3. PAUF Knockdown Inhibits TGF-β-Induced Migration and Invasion in HepG2 Cells

**Figure 4:**
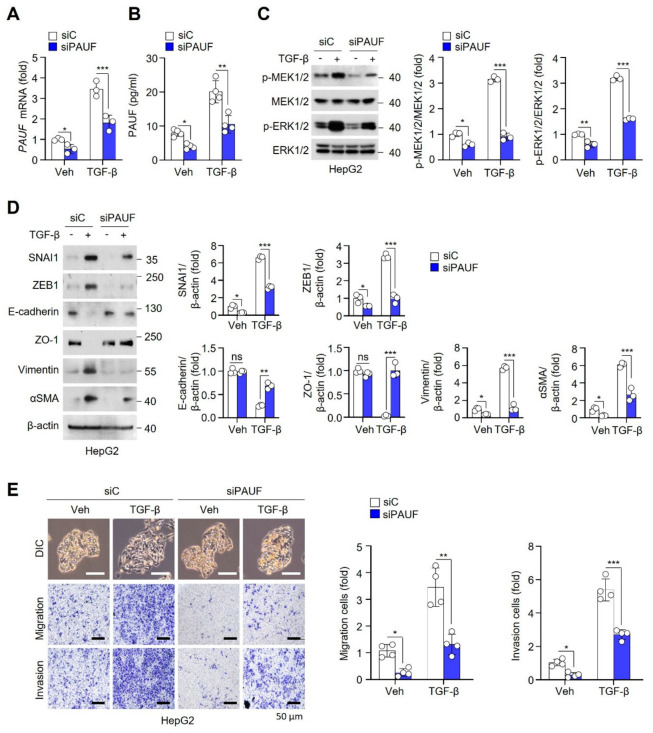
PAUF knockdown inhibits the TGF-β-mediated increase in epithelial–mesenchymal transition (EMT) in HepG2 cells. HepG2 cells were transfected with scramble (Ctrl) or PAUF siRNA and treated with vehicle or TGF-β (10 ng/mL) for (**C**) 1 h or (**A**,**B**,**D**,**E**) 24 h. (**A**,**B**) Successful knockdown of PAUF identified by qPCR (*n* = 3) and sandwich ELISA (*n* = 4). (**C**) Phosphorylation levels of MEK1/2 and ERK1/2 assessed by immunoblotting; densitometric values of phospho-MEK1/2 and -ERK1/2 were normalized to those of total MEK1/2 and ERK1/2 (*n* = 3). (**D**) Expression levels of EMT-related signaling proteins determined by immunoblotting. (**C**,**D**) Densitometric analysis of immunoblot bands was performed using ImageJ software (*n* = 3). (**E**) Cell morphological changes observed using an optical microscope (magnification, 100×), and cell migration and invasion revealed by transwell migration and Matrigel invasion assays (magnification, 40×). Scale bar, 50 μm. Cell migration and invasion were quantified in four fields per sample (*n* = 4). Statistical significance was evaluated using two-way ANOVA, followed by *post hoc* multiple comparisons test with Bonferroni correction. All data are presented as the mean ± SD. ns, not statistically significant, * *p* < 0.05, ** *p* < 0.01, *** *p* < 0.001.

To explore whether TGF-β-induced PAUF expression contributes to EMT, we first examined the effects of PAUF knockdown in the TGF-βRI/II–Smads signaling-positive HCC cell line HepG2 stimulated with vehicle or TGF-β. PAUF knockdown cells were successfully generated, as confirmed by qRT-PCR and sandwich ELISA (Figure 4A,B). The Smad-independent MEK-ERK signaling pathway plays a pivotal role in TGF-β-mediated cancer progression through EMT promotion [21,31] and is required for migration and invasion in HCC cells [23]. Therefore, we evaluated the effects of PAUF knockdown on TGF-β-activated MEK-ERK signaling in HepG2 cells. PAUF knockdown markedly attenuated both basal and TGF-β-induced phosphorylation of MEK1/2 and ERK1/2, whereas control siRNA had no effect (Figure 4C). Previous studies have shown that TGF-β promotes EMT by activating the MEK–ERK signaling pathway, which induces EMT-associated transcription factors, including SNAI1 and ZEB1. This signaling cascade is accompanied by increased expression of mesenchymal markers, such as N-cadherin, vimentin, and α-SMA, and reduced expression of epithelial markers, including E-cadherin, claudins, and ZO-1, thereby enhancing tumor cell motility, invasion, and metastasis [32,33]. Based on these findings, we investigated whether PAUF knockdown affects the expression of TGF-β-regulated EMT-related factors in HepG2 cells. PAUF knockdown markedly attenuated the TGF-β-induced upregulation of SNAI1, ZEB1, vimentin, and α-SMA, while restoring the expression of epithelial markers, including E-cadherin and ZO-1, compared with control siRNA-transfected cells (Figure 4D). Furthermore, under the same experimental conditions, PAUF knockdown markedly decreased TGF-β-induced increases in EMT-associated morphological changes, migration, and invasion, whereas control siRNA transfection had no effect. In addition, PAUF knockdown significantly inhibited basal cell motility in TGF-β-untreated cells (Figure 4E). Taken together, these PAUF knockdown results suggest that PAUF is involved in regulating EMT-associated cell motility, at least in part through MEK–ERK signaling, under both basal and TGF-β-stimulated conditions.

### 2.4. PAUF Overexpression Promotes TGF-β-Mediated EMT-like Phenotypes and Cell Motility in Huh-7 Cells

**Figure 5:**
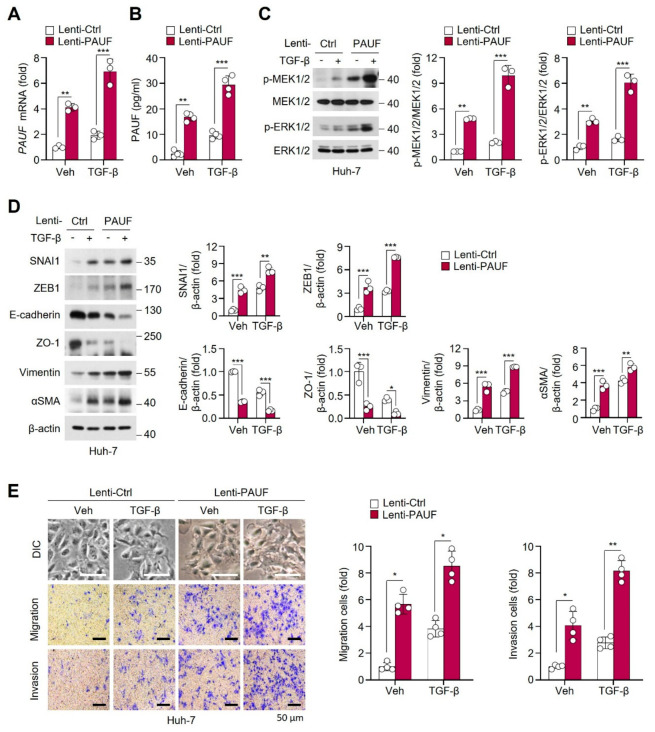
PAUF overexpression augments the TGF-β-induced increase in EMT-like characteristics and cell motility in Huh-7 cells. Huh-7 cells were stably transfected with Lenti-Ctrl or Lenti-PAUF expression vectors, then treated with vehicle or TGF-β for (**C**) 1 h or (**A**,**B**,**D**,**E**) 24 h. (**A**,**B**) Successful ectopic expression of PAUF verified by qPCR (*n* = 3) and sandwich ELISA (*n* = 4). (**C**) Phosphorylated MEK1/2 and ERK1/2 levels determined by immunoblot analysis. (**D**) Expression levels of EMT-related proteins examined by immunoblotting. (**C**,**D**) Densitometry was performed using Image J software (*n* = 3). (**E**) Change in cell morphology observed using an optical microscope (magnification, 100×), and EMT-mediated cell motility analyzed by transwell migration and Matrigel invasion assays (magnification, 40×). Scale bar, 50 μm. Densitometric analysis of cell migration and invasion was performed in four fields per sample (*n* = 4). Statistical significance was calculated using two-way ANOVA, followed by *post hoc* multiple comparisons test with Bonferroni correction. Data are presented as the mean ± SD. * *p* < 0.05, ** *p* < 0.01, *** *p* < 0.001.

We further investigated whether PAUF overexpression regulates the progression of TGF-β-mediated EMT in the low-TGF-βRI/II–Smads signaling HCC cell line (Huh-7). PAUF-overexpressing Huh-7 cells were successfully generated, as confirmed by immunoblotting and sandwich ELISA (Figure 5A,B). We next investigated whether PAUF overexpression modulates EMT-associated MEK–ERK signaling in Huh-7 cells under basal and TGF-β-stimulated conditions. Although TGF-β treatment alone induced only modest increases in MEK1/2 and ERK1/2 phosphorylation, ectopic PAUF expression markedly enhanced MEK–ERK activation in TGF-β-stimulated cells. Notably, PAUF overexpression also increased the phosphorylation levels of MEK1/2 and ERK1/2 under basal conditions (Figure 5C). Compared with control vector (Lenti-Ctrl)-transfected cells, PAUF-overexpressing Huh-7 cells showed increased expression of the MEK–ERK signaling-regulated EMT-inducing transcription factors SNAI1 and ZEB1 under both basal and TGF-β-stimulated conditions. PAUF overexpression also further enhanced the TGF-β-induced upregulation of mesenchymal markers, including α-SMA and vimentin, while further suppressing the expression of epithelial markers, such as E-cadherin and ZO-1, compared with Lenti-Ctrl-transfected cells (Figure 5D). Consistently, compared with control cells, PAUF-overexpressing Huh-7 cells exhibited EMT-related spindle-shaped morphology and increased cell motility, including migration and invasion, even in the absence of TGF-β treatment. These biological processes were prominently enhanced by TGF-β stimulation (Figure 5E). These results suggest that both TGF-β-induced PAUF and ectopically overexpressed PAUF contribute to EMT-related phenotypic changes and enhanced cell motility in HCC cells through mechanisms involving MEK–ERK signaling.

### 2.5. PAUF Enhances Lung Metastatic Potential of HCC Cells In Vivo

**Figure 6:**
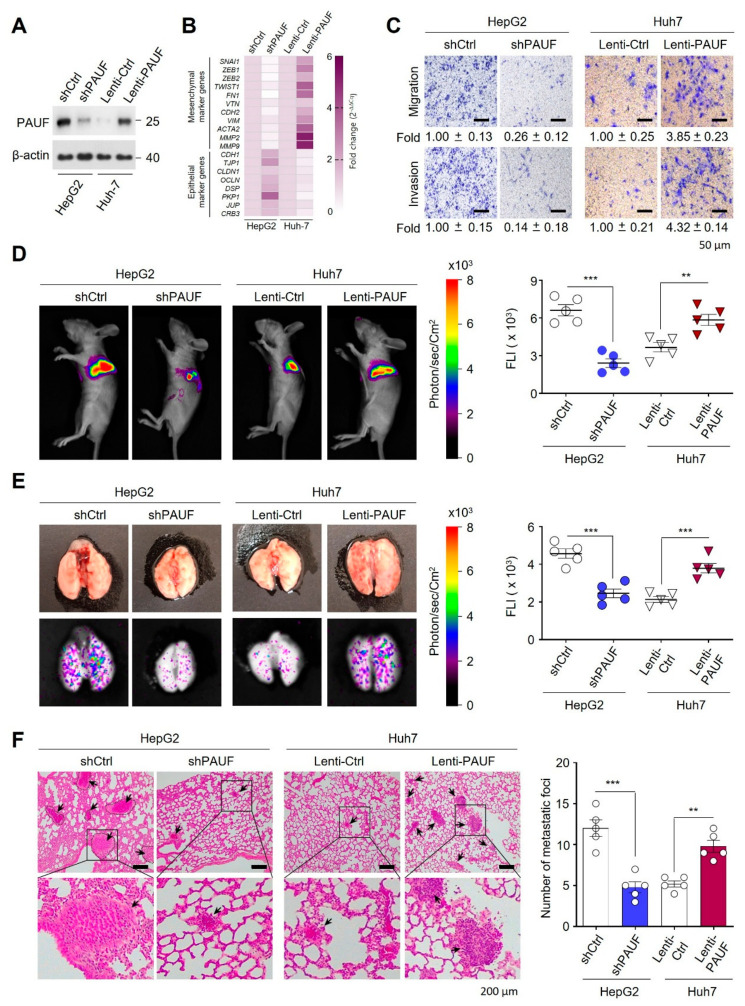
PAUF promotes lung metastasis of HCC cells in vivo. (**A**–**F**) Luciferase-expressing HepG2 and Huh-7 stable cells (Luc-HepG2, Luc-Huh-7) were generated by transfection with a luciferase expression vector (pLenti-Luc), followed by puromycin selection. Luc-HepG2 cells were stably transfected with expression vectors of scrambled (Ctrl) or PAUF shRNA, and Luc-Huh-7 cells were transfected with Lenti-Ctrl or Lenti-PAUF expression vectors. Stable cell lines were then established by puromycin (10 μg/mL) selection. (**A**) Successful knockdown or ectopic expression of PAUF confirmed by immunoblot analysis. (**B**) mRNA expression levels of mesenchymal and epithelial marker genes determined by qPCR (*n* = 3). (**C**) Cell motility of PAUF-knockdown HepG2 and PAUF-overexpressing Huh-7 cells, evaluated using transwell migration and Matrigel invasion assays. Scale bar, 50 μm; magnification, 40×. Densitometric analysis of migrated and invading cells was performed in four fields per sample (*n* = 4). (**D**–**F**) For lung metastatic imaging analysis, a lung metastasis model was established in 6-week-old BALB/c-nude mice (*n* = 5 mice per group). Mice were intravenously injected with PAUF knockdown Luc-HepG2 or PAUF-overexpressing Luc-Huh-7 cells (2 × 10^6^ cells suspended in 200 μL PBS). (**D**,**E**) Lung metastasis visualized using the IVIS Lumina II system, showing representative images of a mouse in each group, analyzed by bioluminescent intensity. (**F**) Representative hematoxylin and eosin-stained lung sections indicating metastatic foci (indicated by arrow). Scale bar, 200 μm; magnification, 10×. Right-hand panels show the number of metastasis foci (*n* = five mice per group). Statistical significance was evaluated using two-way ANOVA followed by *post hoc* multiple comparisons test with Bonferroni correction. Data are presented as the mean ± SD. ** *p* < 0.01, *** *p* < 0.001.

To further explore whether PAUF promotes lung metastasis of HCC cells, PAUF-knockdown HepG2 and PAUF-overexpressing Huh-7 cells were generated using luciferase-expressing HepG2 and Huh-7 (Luc-HepG2, Luc-Huh-7) stable cells. Successful knockdown or ectopic expression of PAUF was confirmed by immunoblotting (Figure 6A). To validate EMT processes in the stable cell lines, we next examined the regulatory effects of PAUF on the mRNA expression of EMT-related marker genes in PAUF-knockdown HepG2 and PAUF-overexpressing Huh-7 cells. PAUF knockdown markedly reduced the basal expression levels of mesenchymal marker genes but increased the expression of epithelial marker genes compared to those in control shRNA-transfected HepG2 cells. Conversely, PAUF overexpression significantly enhanced the expression of mesenchymal marker genes and decreased the basal expression levels of epithelial marker genes compared with those in Lenti-Ctrl-transfected Huh-7 cells (Figure 6B). Moreover, under the same experimental conditions, PAUF-knockdown HepG2 cells exhibited significantly decreased cell migration and invasion compared with control shRNA-transfected HepG2 cells. In contrast, PAUF-overexpressing Huh-7 cells showed strongly increased cell migration and invasion compared with Lenti-Ctrl-transfected cells (Figure 6C). Next, to evaluate the function of PAUF in vivo, PAUF-knockdown HepG2 and PAUF-overexpressing Huh-7 cells were injected into mice via the tail vein to establish a lung metastasis model. The group injected with PAUF-knockdown HepG2 cells showed markedly reduced lung metastasis compared with the group injected with control HepG2 cells. In contrast, the group receiving PAUF-overexpressing Huh-7 cells exhibited significantly enhanced pulmonary metastasis relative to the group injected with control Huh-7 cells (Figure 6D,E). Quantification of lung metastatic foci by hematoxylin and eosin staining yielded consistent results (Figure 6F). Taken together, our findings indicate that PAUF plays a pivotal role in promoting lung metastasis of HCC cells.

## 3. Discussion

In this study, we examined whether elevated PAUF expression contributes to EMT induction, enhanced migration, and lung metastasis, and whether PAUF regulated by TGF-β downstream signaling further promotes EMT-associated motility in HCC cells. Thus, we investigated the functional significance of differential basal PAUF expression between HepG2 and Huh-7 cells, rather than focusing solely on the higher basal migratory capacity of HepG2 cells compared with Huh-7 cells. HepG2 and Huh-7 are widely used HCC cell lines for evaluating migration, invasion, and EMT-associated signaling [34]. Although both cell lines can acquire enhanced migratory and invasive phenotypes in response to pro-metastatic stimuli, such as TGF-β [35], their basal metastatic potential is generally considered relatively low compared with highly metastatic HCC cell lines, such as MHCC97L, MHCC97H, and HCCLM3 [36].

Our results showed high expression of TGF-βRI and TGF-βRII in HepG2 cells, but low expression of TGF-βRI and TGF-βRII in Huh-7 cells (Figure 1B and Figure S1). These results are consistent with analysis of the Human Protein Atlas (https://www.proteinatlas.org) database, as well as previous research [30]. Moreover, our results showed that the TGF-β-induced activation of the TGF-βRI/II-Smad signaling pathway (“canonical”; also known as the Smad-dependent signaling pathway) played a critical role in regulating PAUF expression in HepG2 and Huh-7 cell lines. TGF-β facilitates HCC progression by promoting EMT mediated by canonical SMAD2/3 signaling, as well as non-canonical pathways, such as the MEK–ERK signaling cascade [37,38]. Activation of TGF-β-mediated MEK–ERK signaling enhances EMT-related gene expression and morphological changes, leading to enhanced tumor cell motility, invasiveness, and metastatic potential [39]. Our findings strongly support the regulatory mechanism identified in our previous study, whereby the TGF-β-mediated activation of Smads increased PAUF expression in the PDAC cell line Panc-1, which is crucial for promoting cell migration and invasion through activation of the MEK–ERK signaling pathway [29]. In addition to the canonical Smad-dependent pathway, TGF-β also activates non-Smad signaling pathways, including JNK, p38 MAPK, and ERK1/2 [40]. These pathways regulate downstream transcription factors, such as AP-1 and CREB. JNK and ERK1/2 signaling can enhance AP-1 activity through c-Jun phosphorylation and c-Fos induction, whereas p38 MAPK and ERK1/2 may contribute to CREB activation [41,42,43,44]. Notably, we used the ALGGEN PROMO program 3.0.2 to identify putative binding sites for AP-1 and CREB within the −1.7 kb region of the PAUF promoter. A putative AP-1-responsive element (ARE; 5′-TGACTCCAG-3′) was predicted at −395/−386 bp, and a putative CREB-responsive element (CRE; 5′-GCTACGTCA-3′) was predicted at −1226/−1217 bp. These findings suggest that AP-1 and CREB, as downstream transcription factors of TGF-β-induced non-Smad signaling, may also participate in PAUF transcriptional regulation. Therefore, future studies should investigate whether AP-1 and CREB directly bind to the PAUF promoter and contribute to TGF-β-induced PAUF expression. Elucidating these mechanisms may provide important insights into the complex transcriptional regulation of PAUF and facilitate the development of therapeutic strategies targeting TGF-β-induced, PAUF-mediated cancer metastasis.

In the present study, PAUF knockdown attenuated EMT-associated migration and invasion in HepG2 cells under both basal and TGF-β-stimulated conditions, accompanied by reduced MEK–ERK signaling activation. Conversely, ectopic PAUF expression enhanced migratory and invasive phenotypes in Huh-7 cells, even in the absence of TGF-β stimulation, and these effects were further enhanced by TGF-β treatment. These results suggest that secreted PAUF may enhance EMT-associated HCC migration and invasion through activation of TLR4 signaling. PAUF induces pancreatic cancer metastasis through activation of TLR4-mediated signaling [28]. Furthermore, activation of TLR4 signaling can activate an MEK–ERK signaling cascade, thereby promoting cancer progression and metastasis [45,46,47]. Upon TLR4 stimulation, MEK1/2 and ERK1/2 are phosphorylated, leading to the induction of EMT-associated transcription factors (such as SNAI1, SLUG, and ZEB1/2), the upregulation of mesenchymal markers, and the downregulation of epithelial markers. These processes promote cancer cell motility and invasiveness within the tumor microenvironment. Taken together, TLR4-mediated activation of the MEK–ERK signaling cascade serves as a pivotal enhancer of cancer metastasis by integrating intrinsic tumor cell plasticity with microenvironmental reprogramming [47,48]. Moreover, TLR4 is endogenously expressed in both HepG2 and Huh-7 HCC cell lines [49,50].

In addition to TLR4 signaling, aberrant activation of receptor tyrosine kinases, including EGFR, VEGFR2/3, and PDGFR-β, has also been associated with EMT-mediated cancer cell migration and invasion. These receptor tyrosine kinase pathways may contribute to tumor progression through dysregulated downstream MEK–ERK signaling [51,52,53,54]. However, no studies have determined a direct association between these receptors and TGF-β-induced PAUF in HCC cells. Thus, further investigation is required to elucidate the precise regulatory mechanisms underlying EMT progression, particularly those involving potential interactions between TGF-β-induced PAUF and TLR4 or receptor tyrosine kinases.

Consistent with the in vitro experimental results, our in vivo analyses further demonstrated the functional relevance of PAUF in metastatic progression. PAUF-knockdown HepG2 cells showed markedly diminished lung metastasis compared with control cells, whereas PAUF-overexpressing Huh-7 cells exhibited a pronounced increase in pulmonary metastasis. These findings indicate that PAUF is a key determinant of the metastatic capacity of HCC cells in vivo.

Biologically and mechanistically, our findings suggest that PAUF plays a functional role in HCC metastatic progression by linking TGF-β signaling to EMT-associated cellular plasticity and metastatic competence. In the present study, TGF-β induced PAUF expression through the TGF-β receptor–Smad signaling pathway, and increased PAUF expression promoted EMT-related molecular alterations as well as enhanced the migratory and invasive capacities of HCC cells. These phenotypic changes were associated with activation of the MEK–ERK signaling pathway, suggesting that PAUF may function as a downstream effector that connects TGF-β signaling to EMT-driven invasive and metastatic behavior. Consistent with these in vitro findings, modulation of PAUF expression significantly affected lung metastatic colonization in vivo, as PAUF knockdown attenuated pulmonary metastasis of HepG2 cells, whereas PAUF overexpression enhanced lung metastatic burden in Huh-7 cells. Thus, the TGF-β–PAUF–MEK/ERK axis provides a mechanistic framework for understanding how HCC cells acquire EMT-associated invasive properties and metastatic potential. In the context of lung metastasis, PAUF may facilitate critical phases of the metastatic cascade, including the acquisition of EMT-like plasticity, enhanced invasive capacity, and colonization of the distant pulmonary niche. These findings have translational implications, suggesting that PAUF, or the PAUF–MEK/ERK signaling axis, may represent a potential biomarker and therapeutic target for suppressing EMT-driven invasion and distant metastasis in HCC. Nevertheless, although our findings demonstrate the functional relevance of the TGF-β–PAUF axis in HCC cell models and experimental lung metastasis, histological validation of this pathway remains to be performed. Immunohistochemical analysis of TGF-βRI/II, phosphorylated Smad2/3, PAUF, and EMT-related markers in primary HCC tissues and metastatic lesions would further strengthen the tissue-level relevance and clinical significance of this pathway.

Beyond the genetic and molecular mechanisms described in the present study, HCC progression should also be considered within the broader context of a dynamically evolving tumor ecosystem. Tumors are increasingly recognized as complex ecological systems composed of malignant cells, stromal cells, immune cells, extracellular matrix components, and soluble factors that continuously interact and co-evolve within the tumor microenvironment [55]. In this context, EMT is not simply a fixed molecular program, but rather represents a plastic phenotypic transition that enables cancer cells to adapt to competitive and selective pressures during tumor evolution [56]. The TGF-β–PAUF axis identified in this study may contribute to such EMT-associated phenotypic plasticity by promoting migratory and invasive traits that facilitate metastatic progression. Thus, PAUF-mediated EMT-like changes may represent an adaptive cellular response that enhances the fitness of HCC cells within the evolving tumor ecosystem.

In addition, TGF-β signaling is known to exert context-dependent and stage-dependent effects during cancer progression. During early tumorigenesis, TGF-β can function as a tumor suppressor by inhibiting cell proliferation and inducing apoptosis, whereas in advanced tumors it often promotes EMT, invasion, immune evasion, and metastatic dissemination [57]. In the present study, we focused on the pro-metastatic function of the TGF-β–PAUF axis in HCC cell models, particularly in relation to EMT-associated migration, invasion, and lung metastatic colonization. However, we did not directly examine whether a stage-dependent functional switch of TGF-β occurs during HCC progression, nor did we investigate multicellular tumor ecosystem dynamics or spatial interactions within the tumor microenvironment.

Several methodological and experimental limitations should also be considered when interpreting the present findings. First, although the tail-vein injection lung metastasis model is a useful approach for assessing the lung colonization capacity of HCC cells, it does not fully reproduce the complete process of spontaneous metastasis, including primary tumor formation, local invasion, intravasation, and interactions with the liver tumor microenvironment [58]. Thus, the pulmonary metastatic lesions observed in this model should be interpreted primarily as evidence of metastatic colonization rather than as a complete representation of spontaneous metastasis from a primary liver tumor. Second, because BALB/c nude mice are athymic immunodeficient animals with impaired T-cell-mediated immunity, this model does not fully account for the contribution of host anti-tumor immune responses to HCC progression and metastasis [59]. In addition, the use of selected HCC cell lines may limit the generalizability of our findings to other HCC cell types or clinically heterogeneous disease settings.

Accordingly, future studies incorporating clinical HCC specimens and metastatic tissues, stage-defined patient samples, additional HCC cell lines, orthotopic or spontaneous metastasis models, immune-competent or humanized mouse models, patient-derived HCC models, and single-cell or spatial profiling approaches will be important to validate the relationship among TGF-β/Smad signaling activation, PAUF expression, EMT-associated metastatic progression, and tumor ecosystem dynamics. These approaches will further clarify whether the biological role of the TGF-β–PAUF axis differs between early and advanced stages of HCC and strengthen the biological, translational, and ecological relevance of this pathway in HCC progression and metastasis.

Collectively, our findings demonstrate that PAUF plays a pivotal role in the lung metastatic potential of HCC cells and suggest that targeting PAUF may represent a promising therapeutic strategy for inhibiting metastatic progression in HCC.

## 4. Materials and Methods

### 4.1. Reagents and Chemicals

Antibodies against TGF-βRI (cat# sc-518018; 1:1000), SNAI1 (cat# sc-271977; 1:1000), ZEB1 (cat# sc-515797; 1:1000), α-smooth muscle actin (α-SMA; cat# sc-53142; 1:2000), ZO-1 (cat# sc-33725; 1:1000), vimentin (cat# sc-373717; 1:1000), SMAD4 (cat# sc-7966; 1:1000), and β-actin (cat# sc-47778; 1:3000) were obtained from Santa Cruz Biotechnology (Dallas, TX, USA). Antibodies against p-MEK1/2 (cat# 9121S; 1:1000), MEK1/2 (cat# 9122S; 1:1000), p-ERK1/2 (cat# 9101S; 1:1000), ERK1/2 (cat# 9102S; 1:1000), p-SMAD2 (cat# 3108S; 1:1000), p-SMAD3 (cat# 9520S; 1:1000), SMAD2/3 (cat# 8685S; 1:1000), and E-cadherin (cat# # 3195S; 1:1000) were purchased from Cell Signaling Technology (Beverly, MA, USA). Antibodies against TGF-βRII (cat# ab259360; 1:2000) were purchased from Abcam (Cambridge, UK), and those against PAUF (cat# MAB7777; 1:1000) were obtained from R&D systems, Inc. (Minneapolis, MN, USA). TGF-βRI kinase inhibitor SB-431542 and recombinant human TGF-β were purchased from Sigma-Aldrich (St. Louis, MO, USA) and R&D systems, respectively. Smad2/3 siRNA (cat# sc-37238), ZG16B/PAUF siRNA (cat# sc-93479), control siRNA (cat# sc-37007), ZG16B/PAUF shRNA plasmid (cat# sc-93479-SH), and control shRNA plasmid (cat# sc-108060) were purchased from Santa Cruz Biotechnology. TGF-βRI (cat# SR322023) and TGF-βRII (cat# SR322024) siRNA oligo duplexes were obtained from OriGene Technologies, Inc. (Rockville, MD, USA). pCMV6-XL5 (cat# PCMV6XL5), pCMV-TGF-βRI (cat# SC108520), pCMV-TGF-βRII (cat# SC302306), pLenti-control (cat# PS100092), and pLenti-PAUF (cat# RC202244L3), and pLenti-Luc (cat# PS100138) vectors were obtained from OriGene Technologies, Inc.

### 4.2. Cell Culture

Human HCC cell lines HepG2 (cat. no. HB-8065; RRID:CVCL_0027) was purchased from the American Type Culture Collection (ATCC; Manassas, VA, USA), and Huh-7 (cat. no. 60104; RRID:CVCL_0336) and SNU-878 (cat. no. 00878; RRID:CVCL_5102) were obtained from Korean Cell Line Bank (KCLB; Jongno-gu, Seoul, Republic of Korea). HepG2 and Huh-7 lines were cultured in Dulbecco’s modified Eagle’s medium (DMEM; Gibco, Carlsbad, CA, USA). SNU-878 cell line was maintained in RPMI-1640 medium (Corning Inc., Corning, NY, USA). All media were supplemented with 10% fetal bovine serum (FBS; Gibco) and 1% penicillin/streptomycin (Gibco) at 37 °C in a humidified environment consisting of 95% air and 5% CO_2_. All cell lines were tested for mycoplasma contamination before use in experiments using the e-Myco™ Mycoplasma PCR Detection Kit (ver. 2.0; cat. no. 25235; iNtRON Biotechnology, Seongnam, Republic of Korea), and only mycoplasma-negative cells were used.

### 4.3. Immunoblotting

Cell lysates were obtained according to previously reported procedures [60,61]. Protein concentrations were determined using a BCA protein assay. For immunoblot analysis, 30–50 µg of total protein was combined with 5× loading buffer and denatured at 100 °C for 10 min. The samples were separated on 8–15% sodium dodecyl sulfate–polyacrylamide gels and subsequently transferred onto polyvinylidene fluoride (PVDF) membranes using a transfer device. After transfer, the membranes were blocked for 1 h with either 3% BSA or 5% skim milk prepared in Tris-buffered saline (T&I, Seoul, Republic of Korea) containing 0.1% Tween-20 (Sigma-Aldrich). The membranes were then incubated with the indicated primary antibodies, as described previously [62]. Detailed information on the antibodies used in this study is provided in Section 2.1. Immunoreactive band intensities were measured by densitometric analysis using ImageJ software version 1.54i (National Institutes of Health, Bethesda, MD, USA; accessed on 3 March 2024). Statistical analyses of the quantified data were performed using GraphPad Prism version 9.0 (GraphPad Software Inc., San Diego, CA, USA.

### 4.4. Immunocytochemistry

To assess TGF-β-induced PAUF expression in HepG2 and Huh-7 cells, cells were seeded on glass slides in 12-well plates and cultured for 24 h until they reached 60–70% confluence. After TGF-β treatment, the cells were fixed with 3.7% formaldehyde and permeabilized with 0.1% saponin solution. The slides were then washed twice with phosphate-buffered saline (PBS), blocked with 3% BSA for 2 h, and incubated overnight at 4 °C with mouse monoclonal anti-PAUF antibody (R&D Systems, Minneapolis, MN, USA; 1:500). After washing with PBS, the slides were incubated with Alexa Fluor 488 goat anti-mouse IgG secondary antibody (Thermo Fisher Scientific, Waltham, MA, USA; 1:1000) at 4 °C for 2 h. To investigate the effects of TGF-βRI/II and Smad2/3 knockdown on TGF-β-induced PAUF expression in HepG2 cells, the cells were transfected with scramble control siRNA, TGF-βRI/II siRNA, or Smad2/3 siRNA at a final concentration of 100 nM. The transfected cells were then seeded on glass slides in 12-well plates and cultured until they reached 60–70% confluence. The transfected cells were treated with vehicle or TGF-β (10 ng/mL) for 24 h, followed by fixation with 3.7% formaldehyde and permeabilization with 0.1% saponin solution. The slides were rinsed twice with PBS and blocked with 3% BSA for 2 h. Subsequently, the slides were incubated overnight at 4 °C with mouse monoclonal anti-PAUF antibody (1:500), followed by PBS washing and incubation with Alexa Fluor 488 goat anti-mouse IgG secondary antibody (1:1000) at 4 °C for 2 h. To evaluate whether SB-431542 (Sigma-Aldrich) inhibits TGF-β-induced PAUF expression through the TGF-βRI/II–Smad2/3 signaling cascade, HepG2 cells were pretreated with SB-431542 (10 μM) for 1 h before stimulation with vehicle or TGF-β (10 ng/mL) for 24 h. After treatment, the cells were fixed with 3.7% formaldehyde, permeabilized with 0.1% saponin solution, and washed twice with PBS. The slides were then blocked with 3% BSA for 2 h and incubated overnight at 4 °C with mouse monoclonal anti-PAUF antibody (1:500). Following two washes with PBS, the slides were incubated with Alexa Fluor 488 goat anti-mouse IgG secondary antibody (1:1000) at 4 °C for 2 h. To assess the effect of ectopic TGF-βRI/II expression on PAUF expression, Huh-7 cells were transfected with pCMV6 mock vector or TGF-βRI/βRII expression vector (pTGF-βRI/II). The transfected cells were seeded on glass slides in 12-well plates, cultured until they reached 60–70% confluence, and subsequently treated with vehicle or TGF-β (10 ng/mL) for 24 h. Subsequent immunofluorescence staining procedures were conducted using the same methods described above. For nuclei staining, the slides were further incubated with 1 µg/mL 4ʹ,6-diamidino-2-phenylindole (DAPI; Sigma-Aldrich) for 15 min at room temperature. After mounting with Faramount Aqueous Mounting Medium (Dako, Glostrup, Denmark), slide images were observed using a Zeiss LSM710 confocal microscope (Carl ZEISS, Berlin, Germany) at 400× magnification. Fluorescence intensities were analyzed using ImageJ software.

### 4.5. Chromatin Immunoprecipitation (Chip) Assay

HepG2 cells were either pretreated with SB-431542 (10 μM) for 1 h or transfected with the indicated siRNAs for 24 h, followed by stimulation with vehicle or TGF-β (10 ng/mL) for 1 h. For ectopic receptor expression experiments, Huh-7 cells were transfected with pCMV6 mock vector or pTGF-βRI/II expression vectors (pCMV-TGF-βRI and pCMV-TGF-βRII) and subsequently treated with vehicle or TGF-β for 1 h. Chromatin immunoprecipitation (ChIP) assays were conducted using a Chromatin Immunoprecipitation Assay Kit (Millipore Corporation, Billerica, MA, USA) in accordance with the manufacturer’s protocol. All experimental procedures were performed as previously described [29].

### 4.6. Enzyme-Linked Immunosorbent Assay (Elisa)

To measure secreted PAUF levels, PAUF-knockdown HepG2 cells and PAUF-overexpressing Huh-7 cells were cultured in Dulbecco’s modified Eagle’s medium with or without TGF-β (10 ng/mL) for 24 h. The culture supernatants were then collected, concentrated using Amicon Ultra-15 centrifugal filter units (Millipore, Burlington, MA, USA), and subjected to enzyme-linked immunosorbent assay (ELISA) for quantification of secreted PAUF. Plates were coated with anti-PAUF antibody (5 µg/mL) for 24 h at room temperature and subsequently incubated with concentrated culture supernatants for 2 h at 37 °C. After incubation, biotin-conjugated PAUF detection antibody (250 ng/mL) was added and incubated for 90 min at 37 °C, followed by treatment with streptavidin-HRP (1:5000) for 30 min at 37 °C. Absorbance was measured at 450 nm using a BioTek Epoch 2 Microplate Spectrophotometer (BioTek Instruments, Inc., Winooski, VT, USA). All procedures were performed as previously described [24,28,29].

### 4.7. Reverse Transcription PCR (RT-PCR) and Quantitative Real-Time PCR (qRT-PCR)

Total RNA was isolated from cultured cells using TRIzol reagent (Invitrogen, Carlsbad, CA, USA). For RT-PCR analysis, 1 μg of total RNA was reverse transcribed into cDNA using M-MLV Reverse Transcriptase (Promega, Madison, WI, USA) according to the manufacturer’s instructions. The relative mRNA levels of PAUF and GAPDH were then examined by RT-PCR using gene-specific primers, and the primer sequences are listed in Table 1. For quantitative mRNA expression analysis, cDNA was generated from 0.5 μg of total RNA using ReverTra Ace™ qPCR RT Master Mix with gDNA Remover (Toyobo, Osaka, Japan), following the manufacturer’s recommended protocol. qRT-PCR was performed using Power SYBR™ Green PCR Master Mix (Thermo Fisher Scientific, Waltham, MA, USA) and gene-specific primers on a QuantStudio 3 Real-Time PCR System (Thermo Fisher Scientific). The primer sequences used for qRT-PCR are provided in Table 2. Relative mRNA expression levels were calculated using the 2^−∆∆Cq^ method, with GAPDH serving as the internal control. All assays were conducted in triplicate across three independent experiments.

### 4.8. PAUF Promoter-Luciferase Reporter Assay

To assess whether TGF-β-activated Smad proteins directly regulate PAUF transcription in the HCC cell lines HepG2 and Huh-7, we used PAUF promoter–Luc vectors (Smad-binding element SBE*^WT^*, SBE*^Mut^*, SBE*^∆mut^*) generated in our previous study [29]. HepG2 cells were co-transfected with 100 nM scrambled (Ctrl), TGF-βRI/II (TGF-βRI and -βRII), or Smad2/3 siRNAs alone or in combination with 1 μg of PAUF promoter–Luc vector (pLuc–PAUF) and 1 μg of β-gal expression vector (pCMV–LacZ; Clontech, Palo Alto, CA, USA) using Lipofectamine 3000 reagent (Invitrogen). 6 h after transfection, the media were replaced with complete growth media and stabilized for 24 h, then treated with vehicle or TGF-β (10 ng/mL) for 24 h. HepG2 cells co-transfected with pLuc–PAUF and pCMV–LacZ were pretreated with SB-431542 (10 μM) for 1 h, then treated with or without TGF-β (10 ng/mL) for 24 h. HepG2 cells were transfected with scrambled (Ctrl) or TGF-βRI/II siRNAs together with PAUF promoter–Luc vector (Smad-binding element–wild type, SBE*^WT^*, or Smad-binding element-mutants, SBE*^Mut^* and SBE*^∆mut^*) and pCMV–LacZ vector. After 6 h of transfection, the media were replaced with complete growth media, and the cells were stabilized for 24 h, followed by treatment with vehicle or TGF-β (10 ng/mL) for 24 h. Huh-7 cells were transfected with pCMV6 (Mock) or TGF-βRI/βRII (pTGF-βRI/II) vectors together with PAUF promoter–Luc vectors (SBE*^WT^*, SBE*^Mut^*, or SBE*^∆mut^*) and pCMV–LacZ vector. The transfected cells were stimulated with vehicle or TGF-β (10 ng/mL) for 24 h. Luciferase activity was measured using the Luciferase Reporter Assay System (Promega, Madison, WI, USA) according to the manufacturer’s instructions. β-Galactosidase activity was determined using ortho-nitrophenyl-β-galactosidase (Sigma-Aldrich, St. Louis, MO, USA), and luciferase reporter activity was normalized against β-galactosidase activity.

### 4.9. Cell Migration and Invasion Assays

Cell migration and invasion were evaluated using 24-well Transwell^®^ plates equipped with inserts containing 8 µm pore-size membranes (Corning Inc., NY, USA). For invasion assays, the upper chambers of the inserts were precoated with Matrigel^®^ Matrix (Corning), while uncoated inserts were used for migration assays. The lower chamber of transwell plates was filled with 800 µL of culture medium containing 10% fetal bovine serum. HepG2 (5 × 10^4^), Huh-7 (5 × 10^4^), or PAUF knockdown HepG2 (5 × 10^4^) and PAUF-overexpressing Huh-7 (5 × 10^4^) cells were suspended in 200 µL of serum-free medium in the presence or absence of TGF-β (10 ng/mL) and seeded into the upper chambers. The cells were incubated for 24 h at 37 °C to facilitate migration or invasion. After incubation, cells that had migrated or invaded through the membrane were fixed with absolute methanol and stained with 0.1% crystal violet solution. Cells remaining on the inner surface of the upper chamber were carefully removed with cotton swabs. Images of migrated or invaded cells were acquired using an inverted microscope, and cell numbers were quantified from four randomly selected fields per sample using ImageJ software.

### 4.10. In Vivo Experiments

Male BALB/c-nude mice aged 6 weeks were obtained from JA BIO Co. (Suwon, Republic of Korea) and acclimated for 1 week before the experiments. The mice were maintained in a pathogen-free animal facility under a 12 h light–dark cycle. To establish the lung metastasis model, luciferase-expressing Luc-HepG2 or Luc-Huh-7 cells transfected with the indicated vectors were resuspended in PBS at a density of 2 × 10^6^ cells/200 μL PBS. The suspended cells were then intravenously administered to mice via tail-vein injection. 8 weeks after injection, the mice were sacrificed, and lung metastasis was assessed using the IVIS Lumina II imaging system (Caliper Life Sciences, Hopkinton, MA, USA). Paraffin-embedded lung tissue sections were subjected to hematoxylin and eosin staining to assess metastatic foci in the lungs (*n* = 5 mice per group). All animal experiments were reviewed and approved by the Institutional Animal Care and Use Committee of the Scripps Korea Antibody Institute (SKAI-250731-3). The experimental procedures were conducted in compliance with institutional guidelines for animal care.

### 4.11. Statistical Analysis

All statistical analyses were performed using GraphPad Prism version 9.0 (GraphPad Software Inc., San Diego, CA, USA). The number of replicates for each experiment is indicated in the Figure legends, and representative data were obtained from at least three independent experiments. Quantitative results are presented as the mean ± standard deviation. The normality of data distribution was assessed using the Shapiro–Wilk test before applying parametric statistical analyses. For normally distributed data, Statistical significance was assessed using two-way analysis of variance for analyses involving two independent variables, followed by Bonferroni post hoc multiple-comparison tests, where appropriate. When parametric assumptions could not be met or when non-parametric analysis was considered more appropriate, the Wilcoxon rank-sum test, also known as the Mann–Whitney U test, was used for comparisons between two independent groups. No data points were excluded from the analysis unless predefined exclusion criteria were met. A *p*-value of < 0.05 was considered statistically significant.

## 5. Conclusions

In conclusion, the present study demonstrates that PAUF plays a critical role in regulating the lung metastatic potential of HCC cells. Our findings suggest that PAUF contributes to the acquisition of metastatic phenotypes by promoting EMT-associated molecular alterations and enhancing the migratory and invasive capabilities of HCC cells. Notably, modulation of PAUF expression significantly affected lung metastatic colonization in vivo, further supporting its functional importance in HCC progression. Collectively, these results provide evidence that PAUF acts as a pivotal mediator of HCC metastasis and may represent a promising therapeutic target for suppressing metastatic progression and improving clinical outcomes in patients with advanced HCC.

## Figures and Tables

**Table 1:** Sequences of gene-specific primers used for RT-PCR.

Gene	Forward Primer (5′–3′)	Reverse Primer (5′–3′)
*PAUF*	CATGAAATCACAGGGCTGCG	CTGGGGTAGGCAGAGGAGAT
*GAPDH*	ACATGTTCCAATATGATTCCACCC	ATGGACTGTGGTCATGAGTCCTT

**Table 2:** Sequences of gene-specific primers used for qRT-PCR.

Gene	Forward Primer (5′–3′)	Reverse Primer (5′–3′)
*PAUF*	GCACCACTGAAGACTACGACCAT	TGCAGGGTGACTTCCTGGGTATT
*SNAI1*	GAGCTGCAGGACTCTAATCC	ATGAGCATTGGCAGCGAGG
*ZEB1*	CTGCCCAGTTACCCACAATC	AGGGTGGTTCTTGGACTGCA
*ZEB2*	AAGCCTCTGTAGATGGTCCA	ATAATGGCTGTGTCACTGCG
*TWIST1*	ATTCAGACCCTCAAGCTGGC	TCCATCCTCCAGACCGAGAA
*FN1*	GAGAACAGTGGCAGAAGGAA	TTGTAGGACTGGCCAGTAGT
*VTN*	GTACACGGTCTATGACGATG	CTCAGGTTTCAGAACAGGTG
*CDH2*	TCAGTGAAGGAGTCAGCAGA	TCTGGCAAGTTGATTGGAGG
*VIM*	CGCCATCAACACCGAGTTCA	CCTTGAGCTGCTCGAGCT
*ACTA2*	AAGACAGCTACGTGGGTG	GAGCAGGGTGGGATGCT
*MMP2*	AGTGGTCCGTGTGAAGTATG	AAAGTTGTAGGTGGTGGAGC
*MMP9*	ACCAATCTCACCGACAGGCA	CAGGGACAGTTGCTTCTGGA
*CDH1*	TCTGGATAGAGAACGCATTGC	GCTTGTTGTCATTCTGATCGGT
*TJP1*	CATCCACTCTGCTAATGCCT	GGAATGATCAGAAGGCTCTG
*CLDN1*	CAGATCCAGTGCAAAGTCTT	ATACACTTCATGCCAACGGT
*OCLN*	TGGACAGGTATGACAAGTCC	TTCCACATAGTCAGATGGGG
*DSP*	TGAATACAAGCGTCAGGTGC	AGTCACAGAGAGCTCTGAGA
*PKP1*	AACAAGGACCTGTCCTTTGG	CAATGGCCTGGTACTTCTCA
*JUP*	AACCTCTCAGATGTGGCCAC	TGTCAGGTTGGAGAGTGTGC
*CRB3*	CTGGGGGCAAATACAGACCA	AAGGCAGCCAAGAGGGAGAA
*GAPDH*	GGGGCTCTCCAGAACATCAT	GGTCAGGTCCACCACTGACA

## Data Availability

All data supporting the findings of this study are available upon request from the corresponding author.

## Supplementary Materials

The following supporting information can be downloaded at: https://www.mdpi.com/article/10.3390/ijms27146213/s1.

## References

[1] Torre L.A., Bray F., Siegel R.L., Ferlay J., Lortet-Tieulent J., Jemal A. (2015). Global cancer statistics, 2012. CA Cancer J. Clin..

[2] Lei Y., Yee L.W., Ping Z.W. (2015). Target patients for partial hepatectomy and relationship between PLT and prognosis in BCLC B HCC. J. Hepatol..

[3] Critelli R.M., De Maria N., Villa E. (2015). Biology of hepatocellular carcinoma. Dig. Dis..

[4] Li Y., Tang Z.Y., Hou J.X. (2011). Hepatocellular carcinoma: Insight from animal models. Nat. Rev. Gastroenterol. Hepatol..

[5] Yang J., Weinberg R.A. (2008). Epithelial–mesenchymal transition: At the crossroads of development and tumor metastasis. Dev. Cell.

[6] Yeung K.T., Yang J. (2017). Epithelial-mesenchymal transition in tumor metastasis. Mol. Oncol..

[7] Mittal V. (2018). Epithelial mesenchymal transition in tumor metastasis. Annu. Rev. Pathol..

[8] Giannelli G., Koudelkova P., Dituri F., Mikulits W. (2016). Role of epithelial to mesenchymal transition in hepatocellular carcinoma. J. Hepatol..

[9] Xu J., Li X., Yang H., Chang R., Kong C., Yang L. (2013). SIN1 promotes invasion and metastasis of hepatocellular carcinoma by facilitating epithelial-mesenchymal transition. Cancer.

[10] Davis F.M., Stewart T.A., Thompson E.W., Monteith G.R. (2014). Targeting EMT in cancer: Opportunities for pharmacological intervention. Trends Pharmacol. Sci..

[11] Hao Y., Baker D., Ten Dijke P. (2019). TGF-β-mediated epithelial-mesenchymal transition and cancer metastasis. Int. J. Mol. Sci..

[12] Massagué J. (2012). TGFβ signalling in context. Nat. Rev. Mol. Cell Biol..

[13] Morikawa M., Derynck R., Miyazono K. (2016). TGF-β and the TGF-β family: Context-dependent roles in cell and tissue physiology. Cold Spring Harb. Perspect. Biol..

[14] Hata A., Chen Y.G. (2016). TGF-β signaling from receptors to Smads. Cold Spring Harb. Perspect. Biol..

[15] Heldin C.H., Moustakas A. (2016). Signaling receptors for TGF-β family members. Cold Spring Harb. Perspect. Biol..

[16] Massagué J., Sheppard D. (2023). TGF-β signaling in health and disease. Cell.

[17] Aashaq S., Batool A., Mir S.A., Beigh M.A., Andrabi K.I., Shah Z.A. (2022). TGF-β signaling: A recap of SMAD-independent and SMAD-dependent pathways. J. Cell. Physiol..

[18] Seoane J., Gomis R.R. (2017). TGF-β family signaling in tumor suppression and cancer progression. Cold Spring Harb. Perspect. Biol..

[19] Xin X., Cheng X., Zeng F., Xu Q., Hou L. (2024). The role of TGF-β/SMAD signaling in hepatocellular carcinoma: From mechanism to therapy and prognosis. Int. J. Biol. Sci..

[20] Giarratana A.O., Prendergast C.M., Salvatore M.M., Capaccione K.M. (2024). TGF-β signaling: Critical nexus of fibrogenesis and cancer. J. Transl. Med..

[21] Olea-Flores M., Zuñiga-Eulogio M.D., Mendoza-Catalán M.A., Rodríguez-Ruiz H.A., Castañeda-Saucedo E., Ortuño-Pineda C., Padilla-Benavides T., Navarro-Tito N. (2019). Extracellular-signal regulated kinase: A central molecule driving epithelial-mesenchymal transition in cancer. Int. J. Mol. Sci..

[22] Giannelli G., Villa E., Lahn M. (2014). Transforming growth factor-β as a therapeutic target in hepatocellular carcinoma. Cancer Res..

[23] Ji J., Cheng Y., Hao S., Luo Y., Fan J., Lu L. (2015). Blockade of ERK1/2 signaling suppresses TGF-β-induced EMT and invasion in hepatocellular carcinoma cells. Oncotarget.

[24] Kim Y.J., Jiang F., Park J., Jeong H.H., Baek J.E., Hong S.M., Jeong S.Y., Koh S.S. (2022). PAUF as a target for treatment of high PAUF-expressing ovarian cancer. Front. Pharmacol..

[25] Escudero-Paniagua B., Bartolomé R.A., Rodríguez S., De Los Ríos V., Pintado L., Jaén M., Lafarga M., Fernández-Aceñero M.J., Casal J.I. (2020). PAUF/ZG16B promotes colorectal cancer progression through alterations of the mitotic functions and the Wnt/β-catenin pathway. Carcinogenesis.

[26] Choi C.H., Chung J.-Y., Park H.-S., Jun M., Lee Y.-Y., Kim B.-G., Hewitt S.M. (2015). Pancreatic adenocarcinoma up-regulated factor expression is associated with disease-specific survival in cervical cancer patients. Hum. Pathol..

[27] Song J., Lee J., Kim J., Jo S., Kim Y.J., Baek J.E., Kwon E.S., Lee K.P., Yang S., Kwon K.S. (2016). Pancreatic adenocarcinoma up-regulated factor (PAUF) enhances the accumulation and functional activity of myeloid-derived suppressor cells (MDSCs) in pancreatic cancer. Oncotarget.

[28] Youn S.E., Jiang F., Won H.Y., Hong D.E., Kang T.H., Park Y.Y., Koh S.S. (2022). PAUF induces migration of human pancreatic cancer cells exclusively via the TLR4/MyD88/NF-κB signaling pathway. Int. J. Mol. Sci..

[29] Lee M., Ham H., Lee J., Lee E.S., Chung C.H., Kong D.H., Park J.R., Lee D.K. (2024). TGF-β-induced PAUF plays a pivotal role in the migration and invasion of human pancreatic ductal adenocarcinoma cell line Panc-1. Int. J. Mol. Sci..

[30] Serova M., Tijeras-Raballand A., Dos Santos C.D., Albuquerque M., Paradis V., Neuzillet C., Benhadji K.A., Raymond E., Faivre S., de Gramont A. (2015). Effects of TGF-β signalling inhibition with galunisertib in hepatocellular carcinoma models and in ex vivo whole tumor tissue samples from patients. Oncotarget.

[31] Xie L., Law B.K., Chytil A.M., Brown K.A., Aakre M.E., Moses H.L. (2004). Activation of the Erk Pathway is required for TGF-β1-induced EMT in vitro. Neoplasia.

[32] Thiery J.P., Sleeman J.P. (2006). Complex networks orchestrate epithelial-mesenchymal transitions. Nat. Rev. Mol. Cell Biol..

[33] Ikenouchi J., Matsuda M., Furuse M., Tsukita S. (2003). Regulation of tight junctions during the epithelium-mesenchyme transition: Direct repression of the gene expression of claudins/occludin by Snail. J. Cell Sci..

[34] Aden D.P., Fogel A., Plotkin S., Damjanov I., Knowles B.B. (1979). Controlled Synthesis of HBsAg in a Differentiated Human Liver Carcinoma-Derived Cell Line. Nature.

[35] Lin X.L., Liu M., Liu Y., Hu H., Pan Y., Zou W., Fan X., Hu X. (2018). Transforming Growth Factor β1 Promotes Migration and Invasion in HepG2 Cells: Epithelial-to-Mesenchymal Transition via JAK/STAT3 Signaling. Int. J. Mol. Med..

[36] Jiang K., Li W., Shang S., Sun L., Guo K., Zhang S., Liu Y. (2016). Aberrant Expression of Golgi Protein 73 Is Indicative of a Poor Outcome in Hepatocellular Carcinoma. Oncol. Rep..

[37] Xu J., Lamouille S., Derynck R. (2009). TGF-β-induced epithelial-to-mesenchymal transition. Cell Res..

[38] Tumbrink H.L., Heimsoeth A., Sos M.L. (2021). The next tier of EGFR resistance mutations in lung cancer. Oncogene.

[39] Zhang Y.E. (2017). Non-Smad pathways of TGF-β signaling in tumor progression. Nat. Rev. Cancer.

[40] Javelaud D., Mauviel A. (2005). Crosstalk mechanisms between the mitogen-activated protein kinase pathways and Smad signaling downstream of TGF-beta: Implications for carcinogenesis. Oncogene.

[41] Zhang Y.E. (2009). Non-Smad pathways in TGF-β signaling. Cell Res..

[42] Karin M. (1995). The regulation of AP-1 activity by mitogen-activated protein kinases. J. Biol. Chem..

[43] Deak M., Clifton A.D., Lucocq J.M., Alessi D.R. (1998). Mitogen- and stress-activated protein kinase-1, MSK1, is directly activated by MAPK and SAPK2/p38, and may mediate activation of CREB. EMBO J..

[44] Koga Y., Tsurumaki H., Aoki-Saito H., Sato M., Yatomi M., Takehara K., Hisada T. (2019). Roles of cyclic AMP response element binding activation in the ERK1/2 and p38 MAPK signalling pathway in central nervous system, cardiovascular system, osteoclast differentiation and mucin and cytokine production. Int. J. Mol. Sci..

[45] Huang B., Zhao J., Unkeless J.C., Feng Z.H., Xiong H. (2005). TLR4 signaling promotes tumor growth and paclitaxel resistance in ovarian cancer. Cancer Res..

[46] Coventry B.J., Ashdown M.L., Quinn M.A., Markovic S.N., Yatomi-Clarke S.L., Robinson A.P. (2009). CRP identifies homeostatic immune oscillations in cancer patients: A potential treatment targeting tool?. J. Transl. Med..

[47] Kim S.J., Lee S.Y., Lee C., Kim I., An H.J., Kim J.Y., Baek K.H., Kim E.J., Kim J.M., Lee J.B. (2006). Differential expression profiling of genes in a complete hydatidiform mole using cDNA microarray analysis. Gynecol. Oncol..

[48] Fukata M., Chen A., Vamadevan A.S., Cohen J., Breglio K., Krishnareddy S., Hsu D., Xu R., Harpaz N., Dannenberg A.J. (2007). Toll-like receptor-4 promotes the development of colitis-associated colorectal tumors. Gastroenterology.

[49] Hsiao C.C., Chen P.H., Cheng C.I., Tsai M.S., Chang C.Y., Lu S.C., Hsieh M.C., Lin Y.C., Lee P.H., Kao Y.H. (2015). Toll-like receptor-4 is a target for suppression of proliferation and chemoresistance in HepG2 hepatoblastoma cells. Cancer Lett..

[50] Kalanxhi E., Hektoen H.H., Meltzer S., Dueland S., Redalen K.R., Flatmark K., Ree A.H. (2015). Abstract 571: Circulating proteins in response to combined-modality therapy in locally advanced rectal cancer identified by antibody array screening. Cancer Res..

[51] Roberts P.J., Der C.J. (2007). Targeting the Raf-MEK-ERK mitogen-activated protein kinase cascade for the treatment of cancer. Oncogene.

[52] Sebolt-Leopold J.S. (2008). Advances in the development of cancer therapeutics directed against the RAS-mitogen-activated protein kinase pathway. Clin. Cancer Res..

[53] Kohno M., Pouysségur J. (2003). Pharmacological inhibitors of the ERK signaling pathway: Application as anticancer drugs. Prog. Cell Cycle Res..

[54] Sebolt-Leopold J.S., Herrera R. (2004). Targeting the mitogen-activated protein kinase cascade to treat cancer. Nat. Rev. Cancer.

[55] Chen X., Song E. (2022). The theory of tumor ecosystem. Cancer Commun..

[56] Pastushenko I., Blanpain C. (2019). EMT transition states during tumor progression and metastasis. Trends Cell Biol..

[57] Principe D.R., Doll J.A., Bauer J., Jung B., Munshi H.G., Bartholin L., Pasche B., Lee C., Grippo P.J. (2014). TGF-β: Duality of function between tumor prevention and carcinogenesis. J. Natl. Cancer Inst..

[58] Gómez-Cuadrado L., Tracey N., Ma R., Qian B., Brunton V.G. (2017). Mouse models of metastasis: Progress and prospects. Dis. Models Mech..

[59] Olson B., Li Y., Lin Y., Liu E.T., Patnaik A. (2018). Mouse models for cancer immunotherapy research. Cancer Discov..

[60] Lee D.K., Kim J.H., Kim J., Choi S., Park M., Park W., Kim S., Lee K.S., Kim T., Jung J. (2018). REDD-1 aggravates endotoxin-induced inflammation via atypical NF-κB activation. FASEB J..

[61] Kim D., Go S.H., Song Y., Lee D.K., Park J.R. (2024). Decursin induces G1 cell cycle arrest and apoptosis through reactive oxygen species-mediated endoplasmic reticulum stress in human colorectal cancer cells in in vitro and xenograft models. Int. J. Mol. Sci..

[62] Lee K.S., Kim J., Kwak S.N., Lee K.S., Lee D.K., Ha K.S., Won M.H., Jeoung D., Lee H., Kwon Y.G. (2014). Functional role of NF-κB in expression of human endothelial nitric oxide synthase. Biochem. Biophys. Res. Commun..

