# Supplementary material for: Pancreatic Adenocarcinoma Up-Regulated Factor Promotes Epithelial–Mesenchymal Transition and Lung Metastasis in Hepatocellular Carcinoma
Source: Int J Mol Sci. 2026 Jul 12;27(14):6213. doi: 10.3390/ijms27146213 (PMC13409984; doi:10.3390/ijms27146213)
Supplement: Supplementary file 1 [file ijms-27-06213-s001.zip › ijms-4398142-supplementary.pdf]

Pancreatic Adenocarcinoma Up-Regulated Factor Promotes Epithelial–Mesenchymal Transition and Lung Metastasis in Hepatocellular Carcinoma

Supplementary materials

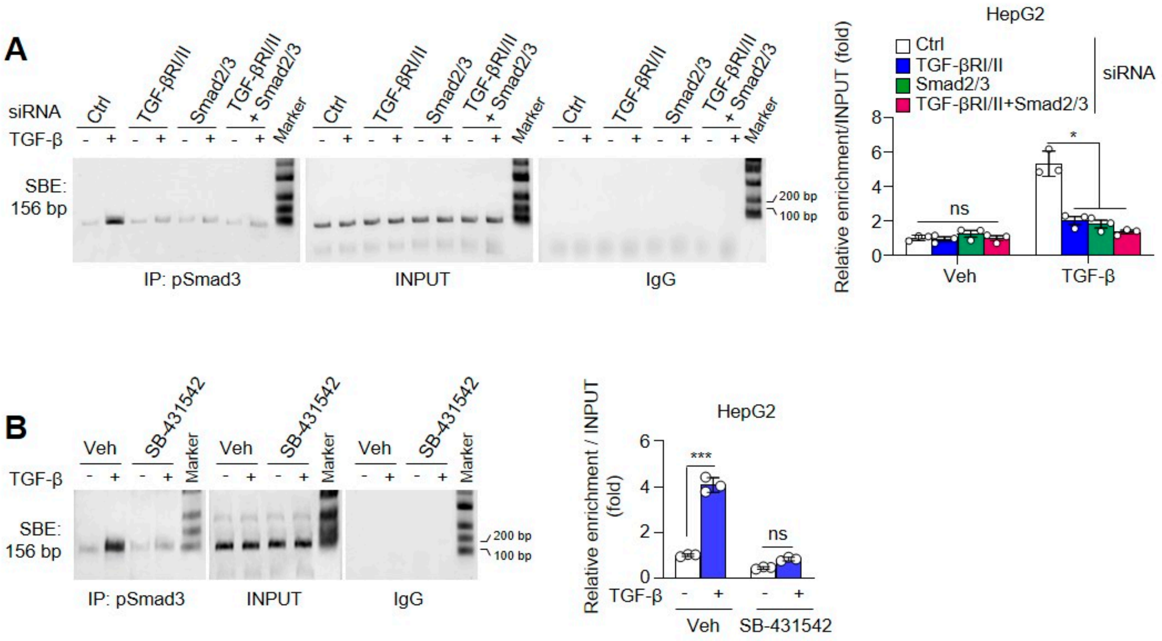

Figure S1. TGF-β-activated Smad3 binds to Smad-binding element (SBE) within the PAUF promoter in HepG2 cells. **(A)** HepG2 cells were transfected with scramble (Ctrl)-, TGF-βRI/II-, or Smad2/3-targeting siRNAs alone or in combination, then treated with or without TGF-β for 1 h to analyze pSmad3 binding to SBE within the PAUF promoter using a chromatin immunoprecipitation (ChIP) assay**. (B)** HepG2 cells were pretreated with SB-431542 (10 μM) for 1 h and subsequently treated with vehicle or TGF-β (10 ng/mL) for 1 h. Binding of pSmad3 to SBE within the PAUF promoter was determined using the ChIP assay. **(A, B)** Fold enrichment of the SBE motif (−804/−801) in the ChIP assay was quantified and normalized to the input control ($n = 3$). Statistical significance was calculated using two-way ANOVA, followed by *post-hoc* multiple comparisons test with Bonferroni correction. Data are presented as the mean ± SD. ns, not statistically significant, *$p < 0.05$, *** $p < 0.001$.
